# A rare case of supinator syndrome caused by osteofibrous dysplasia of the radius

**DOI:** 10.1016/j.jpra.2025.12.023

**Published:** 2025-12-18

**Authors:** Johannes C. Heinzel, Luisa Lange, Julia Wittlinger, Natalie Winter, Vladyslav Kavaka, Adrien Daigeler, Jonas Kolbenschlag, Henrik Lauer

**Affiliations:** a Department of Hand-, Plastic, Reconstructive and Burn Surgery, BG Unfallklinik Tuebingen, University of Tuebingen, Schnarrenbergstraße 95, Tuebingen 72076, Germany; b Department of Neurology and Hertie Institute for Clinical Brain Research (HIH), University of Tuebingen, Hoppe-Seyler-Str. 3, Tuebingen 72076, Germany

**Keywords:** Peripheral nerve, Microsurgery, Nerve compression, Osteofibrous dysplasia, Supinator syndrome

## Abstract

We report the case of a 57-year-old male who presented to our emergency room due to progressive paresis of the muscles innervated by the deep branch of the radial nerve in the right forearm. The patient suffered from osteofibrous dysplasia and an x-ray revealed the polyostotic disease had also affected his radius. High resolution ultrasound revealed that an exostosis of the radius had caused compression of the posterior interosseous nerve unusually distally in the supinator tunnel. The patient underwent nerve decompression and recovered full strength in his wrist-, and finger extensors approximately 9 months postoperatively. This case report illustrates a case of a rare disease, i.e. osteofibrous dysplasia of the radius, in conjunction with the first published report of an unusually located nerve compression syndrome, i.e. supinator syndrome, caused by this very disease.

## Introduction

Osteofibrous dysplasia (OFD) is a rare, self-limiting and benign disease, characterized by fibrous alterations of long bones, most commonly the fibula and tibia. First described in 1921 by Frangenheim^1^ and later characterized by Campanacci in 1976,^2^ OFD accounts for a very small fraction of all primary bone tumors. It predominantly occurs with a slight female preponderance in children and adolescents, with most cases diagnosed before the age of 15. Clinically, patients suffering from OFD present with localized pain, swelling, progressive tibial bowing, or, less frequently, pathological fractures. While often unilateral, bilateral lesions have also been documented. Radiographically, OFD is characterized by an eccentric, cortical-based lytic lesion with a distinct sclerotic rim. Histopathologically, OFD is defined by a fibro-osseous architecture composed of irregular trabeculae of woven bone rimmed by osteoblasts, embedded in a loose fibrous stroma that can assume a storiform pattern. The etiology of OFD remains incompletely understood. Sporadic and familial cases have been reported, with germline or somatic mutations in the MET proto-oncogene emerging as a plausible molecular driver. Management of OFD is typically conservative. Most lesions stabilize or regress with skeletal maturity, and observation with symptomatic treatment (e.g., orthoses for deformity, analgesics for pain) is the standard approach. Surgical intervention is reserved for cases with severe deformity, progressive symptoms, or impending fracture.^3^, ^4^, ^5^ While OFD is generally confined to the skeletal cortex and managed conservatively, extraosseous manifestations, particularly those involving adjacent neurovascular structures, are exceedingly uncommon. To our knowledge, compressive neuropathies in association with OFD have not been described yet. Here, we report the unique case of a patient presenting with radial nerve compression in the forearm caused by OFD of the proximal radius, highlighting both the diagnostic challenges and the therapeutic considerations when a benign fibro-osseous lesion exerts secondary effects on peripheral nerve function.

## Case report

**Figure 1:**
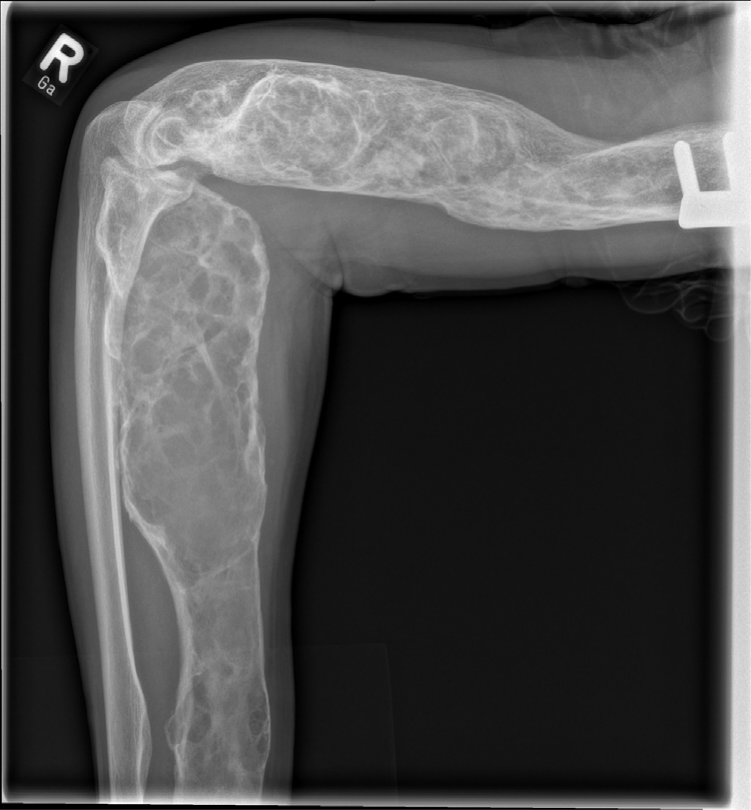
X Ray of the patient’s right upper extremity. The X ray revealed an involvement of the radius and humerus in the context of osteofibrous dysplasia.

The 57-year-old patient was initially admitted to our emergency room and reported paralysis of the right finger extensors that had been present for approximately 3 weeks, initially affecting only the middle, ring, and little fingers, but since 2 days complete paralysis of the index finger and thumb extensors had also occurred. Extension of the right wrist was possible, but strength was reduced to 4/5 according to the MRC scale and radial deviation of the right wrist was apparent during extension. A positive Hoffmann-Tinel sign could be elicited above the course of the radial nerve in the forearm. There were no sensory symptoms regarding the right upper extremity. During anamnesis, the patient reported that he suffered from OFD and a subsequent x-ray (Figure 1) of his right upper extremity revealed polyostotic OFD which had affected both the humerus as well as the radius.

**Figure 2:**
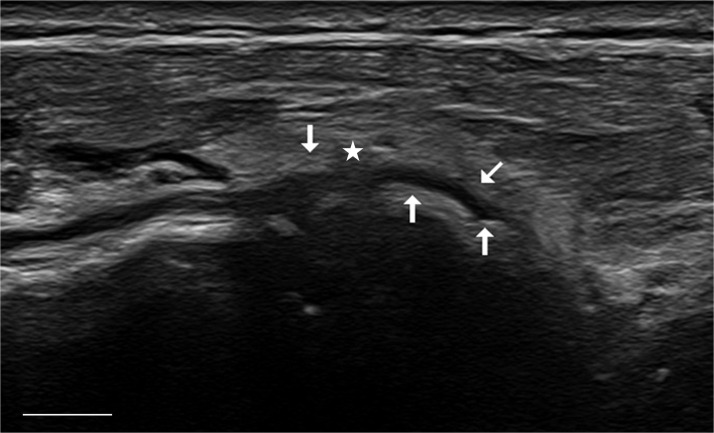
High-resolution ultrasound of posterior interosseus nerve in the distal aspect of the supinator tunnel. The posterior interosseous nerve is depicted in longitudinal view (small arrows) from proximal (left) to distal (right). Note the main compression site of the nerve (star) due to an exostosis of the radius with slight nerve enlargement located proximally. Scale bar: 5 mm.

**Figure 3:**
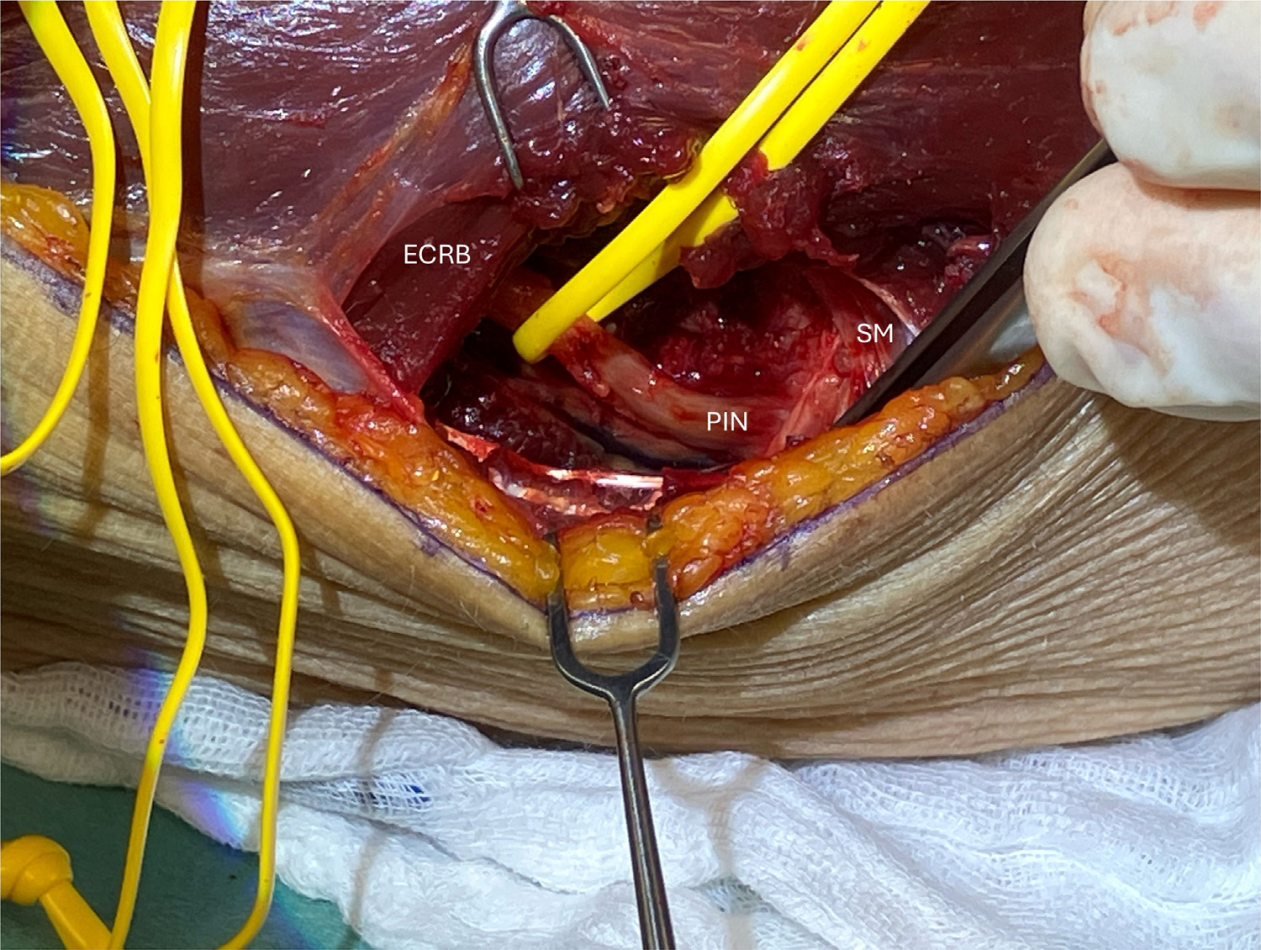
Intraoperative situs of the patient’s right forearm during decompression surgery. PIN: Posterior interosseous nerve. ECRB, extensor carpi radialis muscle; SM, supinator muscle.

The patient was referred to the Department of Neurology for electrodiagnostic testing and high-resolution nerve ultrasound, which were performed approximately 2 weeks later. Nerve ultrasound revealed a normal appearance of the right radial nerve along its entire course in the upper arm. The radial nerve entered the supinator canal without any signs of compression, but the deep branch showed signs of nerve compression with a decrease of nerve diameter. at the distal end of the supinator tunnel due to a small exostosis of the radius. Proximally to the compression site, the deep branch of the radial nerve appeared significantly thickened with a blurred internal structure (Figure 2). More distally there were no additional sign of compression at any point. The electrophysiological findings were also consistent with compression of the right radial nerve on the right in the supinator tunnel. The patient underwent surgery 4 weeks after being examined in our emergency room. A dorsal approach to the proximal forearm was used to explore the radial tunnel. The interval between the extensor carpi radialis muscles and the brachioradialis was developed, allowing identification of the superficial radial nerve and the posterior interosseous. At the level of the middle forearm nerve a bony exostosis had direct contact with the posterior interosseous nerve (PIN), causing the compression neuropathy (Figure 3). Intraoperative nerve stimulation revealed preserved responses in branches to the extensor carpi radialis longus and brevis muscles, whereas the PIN showed markedly reduced motor responses, particularly to the extensor pollicis longus muscle. The Arcade of Frohse was consequently divided, and the PIN was neurolyzed over a long distance and then transposition away from the exostosis, ending the procedure.

**Figure 4:**
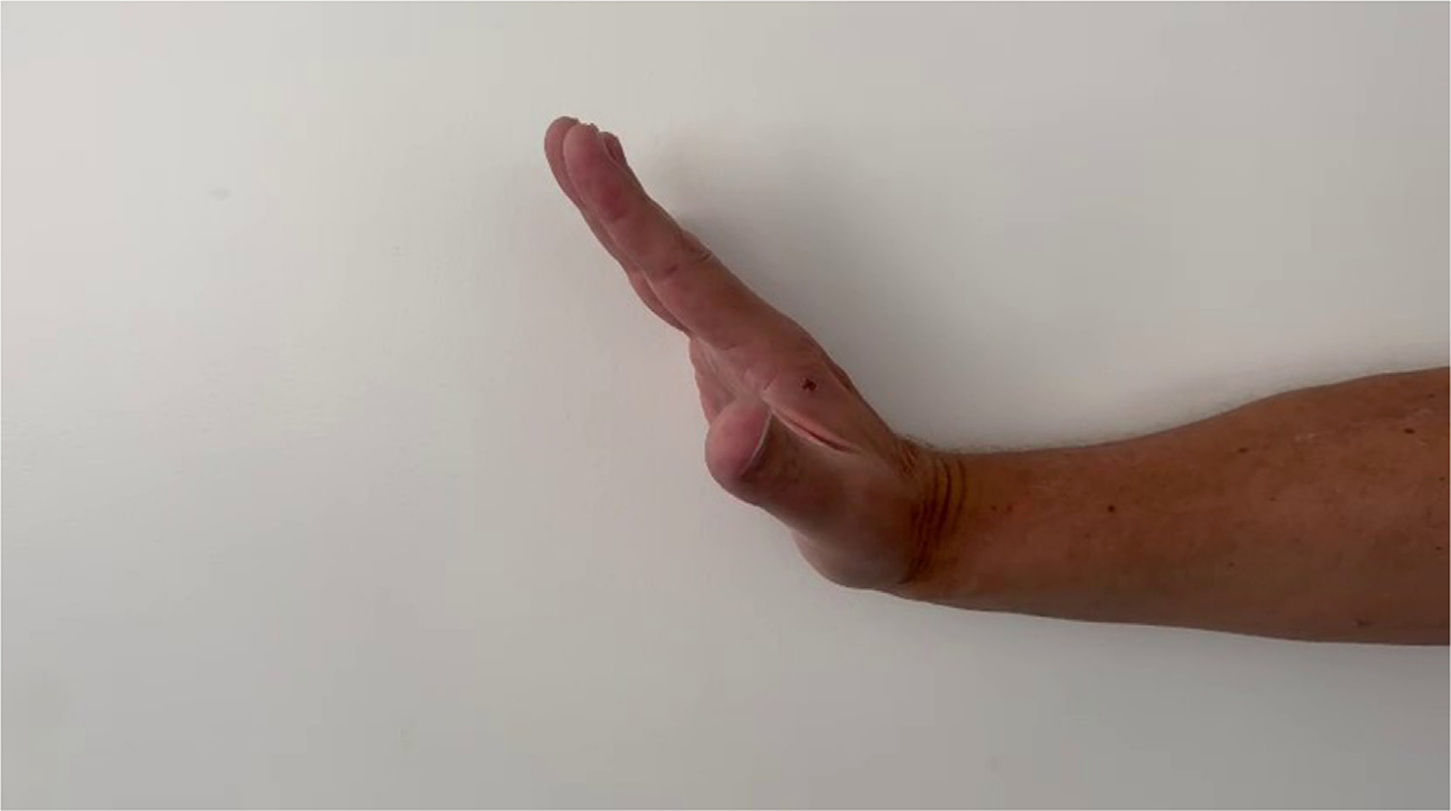
Postoperative function of the patient’s right radial nerve following decompression surgery.

When the patient presented to our outpatient clinic approximately 7 months postoperatively, he was able to fully extend his finger and thumb (Figure 4). While finger extension could be demonstrated with a strength grade of 5/5 according to the MCR scale, thumb extension was slightly weaker with a rating of 4/5. There were no sensory disturbances in the innervation area of the superficial branch of the radial nerve. The patient was fully able to return to work in his profession as an engineer.

## Discussion

Our case report demonstrates a rare case of polyostotic OFD causing posterior interosseus nerve compression in the forearm, a causality which has not been reported before in this context. While radial nerve compression syndromes are no rare diseases^6^^,^^7^ the connection with osteofibrous dysplasia represents an exceptional case. While treatments for OFD include both conservative and operative approaches there is no clear consensus in the literature regarding the actual significance of curettage or wide excision.^3^ While the latter options are generally reserved for cases of severe morbidity with persistent pain, recurrent fractures, and deformity, some authors advocate that growing lesions should be resected due to the risk of these lesions developing into malignancy.^3^^,^^8^ While the underlying OD can be easily diagnosed by means of X-rays or computed tomography,^9^ the correct diagnosis of the associated nerve compression syndrome requires neurophysiological examinations.^6^ In cases like the one at hand, the use of high-resolution nerve ultrasound^10^ is especially recommended to precisely detect the exact location of nerve compression, which was unusually distal in the patient presented in this report. As soon as nerve compression has been identified as cause of the patient’s symptoms we advocate for timely surgical treatment to maximize the chances of *restitutio ad integrum* as was fortunately the case for the patient presented here.

## Authors contributions

J.C.H., L.L., J.W. and N.W. examined the patient in the emergency room and inpatient clinic, documented the clinical results and wrote the first draft of the manuscript. H. L., A.D. and J. K. performed the literature research on the topic and critically revised the manuscript. H.L. and V.K. operated on the patient and critically revised the manuscript. All authors read and approved the final version of the manuscript.

## Ethical approval

Not required.

## Statement of conforming to Helsinki declaration

This work was conducted in accordance with the ethical standards of the institutional and/or national research committee and with the 1964 Helsinki Declaration and its later amendments or comparable ethical standards.

## Funding

None.

## Financial disclosures

None of the authors have any competing financial interest to declare. This research did not receive any grant from funding agencies in the public, commercial, or not-for-profit sectors.

## Declaration of competing interest

The authors declare no conflict of interest.
